# Reply to “Propofol and survival: an updated meta‑analysis of randomized clinical trials”

**DOI:** 10.1186/s13054-023-04484-9

**Published:** 2023-05-17

**Authors:** Manuel Alberto Guerrero Gutierrez, José A. Meade-Aguilar, Diego Escarraman Martinez

**Affiliations:** 1 Department of Bariatric Anesthesia, Baja Hospital and Medical Center, Tijuana, Mexico; 2 Division of Rheumatology, Mayo Clinic, Rochester, MN USA; 3 Department of Anesthesia, National Medical Center “La Raza”, Mexico City, Mexico

**Dear Editor,**

Kotani et al. [1] conducted an updated systematic literature review and meta-analysis of the use of propofol in diverse scenarios. We believe that the information provided is relevant but should be interpreted with caution. One major observation is the lack of defined and clearly stated comparative groups. Propofol is used in a wide range of clinical scenarios and groups of patients. Because of this, multiple comparisons of the use of propofol versus other agents and in different scenarios in clinical trials would be expected. Not stating which are these strategies overshadows the validity of the results. Systematic reviews are intended to answer specific questions based on the PICO framework [2], where C for comparison is a vital component. A clinical question should always have a reliable comparator, whereas in the present study, the comparator was “any strategy besides propofol.” As stated in the Cochrane Handbook for Systematic Reviews of Interventions [2], a well-defined comparator group is preferred in the case of standard meta-analysis, while network meta-analysis may be able to perform multiple comparisons between different treatment groups, every time some assumptions are met. Nonetheless, after looking through the present work and the supplementary material, we did not find any evidence of the comparator groups, severely limiting the capacity of interpretation of the data by the readers, and thus, its clinical applicability.

The variable of time becomes vital all the time that patients with different follow-up periods are being included in the same pool for an effect analysis. A common methodological error is missing different times of follow-up for the calculation of the development of an outcome in a longitudinal fashion. This is the rationale behind using time-to event analyses. In the circumstances in which the time-periods are similar, an assumption of equality can be made, and RRs or ORs can be used. But as in the comparative groups, a median follow-up, and the ranges of follow-up were not stated. Mortality is a variable dependent and affected by time. It lacks all clinical significance attributing a negative mortality effect to a drug without knowing after how much time this effect will be seen.

Currently, a vital part of the results should be in the different scenario of the patients, and this cannot be determined with the results obtained by the meta-analysis. There are certain scenarios in which propofol has been seen to be superior to other agents. The work of Shehabi et al. [3] in 2023 carried out in adults less than 65 years, sedation with a combination of propofol with dexmedetomidine revealed that the incremental dose of propofol was associated with a decreased mortality, while those with dexmedetomidine had an increased mortality 90 days. Based on this, the time hypothesis arises, the variability of the outcomes between studies, the fact of taking too short and/or too long times can alter the interpretation in relation to the main outcome [4]. This study showed a faster induction and movement of the lower body and the patient by achieving a higher recovery area score compared to dexmedetomidine, the importance of this work lies in the fact that it took into account the perspective of patients in sedation of short stay, a rarely explored outcome. Finally, in terms of mortality, Schaefer et al. conducted a study in more than 200,000 patients where increasing propofol dose was associated with reduced odds of 1-year mortality in patients without solid cancer (aOR 0.78; 95% CI 0.71–0.85) [5], it seems that the clinical scenario given by the clinical characteristics of the patient and the propofol dose can play a determining role in terms of outcome.

While this meta-analysis provided valuable insights into the use of propofol, it is important for future studies to address these limitations. The inclusion of specific control groups and the reporting of in RCTs and reporting the different follow-up periods, are crucial to ensure that the findings are accurate and reliable.

## Data Availability

Not applicable.
